# Exploration of degradation pathways of six antibiotics using a novel Co_0.5_Fe_0.5_Fe_2_O₄ nanozyme

**DOI:** 10.1038/s41598-025-23740-2

**Published:** 2025-11-14

**Authors:** Sepideh Ghasemi, Farideh Nabizadeh Chianeh

**Affiliations:** 1 Department of Chemistry, Semnan University, Semnan, Iran; 2 Department of Chemistry, Semnan University, Semnan, Iran

**Keywords:** Antibiotic degradation, Co_0.5_Fe_0.5_Fe_2_O₄ nanozyme, Water treatment, Degradation pathways, Advanced oxidation processes (AOPs), Chemistry, Environmental chemistry, Green chemistry

## Abstract

This study investigates the advanced degradation of six common antibiotics using a novel Co_0.5_Fe_0.5_Fe_2_O₄ nanozyme. Under optimal conditions (pH 7, 0.5 mM H_2_O_2_, 15-min reaction time, and room temperature), the nanozyme demonstrated exceptional catalytic performance, achieved near-complete removal of ciprofloxacin, azithromycin, levofloxacin, moxifloxacin, amoxicillin, and metronidazole. Kinetic studies confirmed its superior catalytic activity, with a Michaelis–Menten constant (*K*_*m*_) of 0.0366 mM and a maximum reaction velocity (*V*_*max*_) of 1.10 × 10⁻4 µM·min⁻1. Mass spectrometry (MS) analysis elucidated the degradation pathways by identifying intermediate compounds and stepwise transformations, while total organic carbon (TOC) analysis confirmed the mineralization of pollutants into CO_2_ and H_2_O. Compared to conventional catalysts, Co_0.5_Fe_0.5_Fe_2_O₄ exhibited superior activity without the need external activation, demonstrating its potential for efficient and sustainable water treatment. These findings are significant for environmental remediation and hold promise for practical applications in pharmaceuticals and wastewater treatment. Moreover, this work provides valuable insights into antibiotic degradation pathways, supporting its potential use in sustainable water treatment strategies.

**Supplementary Information:**

The online version contains supplementary material available at 10.1038/s41598-025-23740-2.

##  Introduction

Antibiotics are among the most significant advancements in modern medicine and veterinary science, crucial for treating bacterial infections and preventing disease spread. However, their widespread and often uncontrolled use has resulted in their accumulation in aquatic environments, raising severe environmental and public health concerns. These persistent contaminants resist conventional water treatment methods due to their stable chemical structures and low biodegradability, which allow them to remain in water systems for prolonged periods^1–5^. Recent studies have reported that antibiotic residues in aquatic systems pose significant risks to both ecosystem integrity and human health and while also contributing to the emergence of antibiotic-resistant bacteria and resistance genes^6^.

Antibiotics are frequently detected globally in surface and groundwater, including in treated wastewater, highlighting the inefficiency of conventional water treatment methods in completely removing these persistent contaminants^7^. Biological treatments often fail due to the recalcitrant nature of many antibiotic compounds, which resist microbial degradation. Similarly, chemical processes such as chlorination and ozonation may result in incomplete degradation and generate toxic by-products that further threaten aquatic ecosystems and human health^8^. Microbial degradation methods are often limited by long reaction times, narrow optimal conditions, and the inability of microorganisms to break down structurally complex antibiotics^8,9^. This inefficiency underscores the importance of advanced catalytic strategies, such as nanozyme-based systems, which can achieve rapid and effective degradation under environmentally relevant conditions.

Beyond limited removal, conventional treatments often suffer from intrinsic drawbacks—electron–hole recombination in photocatalysis; mere phase transfer in adsorption (risking secondary pollution); low efficacy of filtration for dissolved organics; and limited removal of soluble pollutants by coagulation–flocculation^10^. Recent studies further emphasize that these drawbacks often lead to incomplete degradation and secondary pollution, underscoring the need for advanced catalytic oxidation processes^11–13^. Catalysis plays a central role not only in pollutant degradation but also in various industrial and energy-related applications, including hydrogen generation, methane conversion, biomass upgrading, and plastic recycling. These processes are indispensable for the transition to sustainable energy and demonstrate the versatility of catalysis across multiple fields^14^.

The presence of antibiotics in water bodies is particularly alarming as it contributes to the emergence of antibiotic-resistant bacteria, a critical global health challenge. Furthermore, their continued discharge into aquatic ecosystems disrupts microbial communities and facilitates the spread of antibiotic-resistant genes (ARGs), which can transfer to human pathogens and worsen the antibiotic resistance crisis^9^. Fluoroquinolones such as ciprofloxacin are frequently detected in hospital and pharmaceutical effluents and can persist in receiving waters; even at trace concentrations, their presence has been linked to antibiotic resistance development and ecological stress^15^.

Recent advancements in nanotechnology have opened new avenues for addressing these challenges, with promising results in antibiotic degradation and removal^16^. Nanocatalysts, with their high surface area and reactivity, are emerging as powerful tools in advanced oxidation processes (AOPs)—chemical techniques that utilize reactive oxygen species (ROS), such as hydroxyl radicals (·OH), to degrade persistent pollutants^17^. For instance, engineered nanocatalysts such as Sn₃O₄@Au and Ag@ZnIn_2_S₄ have recently shown remarkable efficiency in dye and antibiotic degradation, attributed to defect engineering and synergistic interactions with transition metals^11–13^.Among these, nanozymes, a class of nanomaterials with enzyme-like properties, stand out for their exceptional catalytic efficiency, stability, and ability to function under mild conditions ^18^. Understanding the degradation pathways and ensuring the complete mineralization of these persistent pollutants is essential for advancing sustainable environmental solutions. Sulfate-radical AOPs based on peroxymonosulfate (PMS) are reported to offer higher redox potentials (≈2.5–3.1 V) and longer half-lives (≈30–40 μs) than hydroxyl-radical systems, and PMS is more readily activated than persulfate owing to its asymmetric molecular structure^10,19^. Besides conventional H_2_O_2_-based Fenton or Fenton-like processes, other activation strategies have also been explored to enhance catalytic efficiency. For instance, sodium borohydride (NaBH₄) has been reported to significantly improve electron transfer and suppress charge recombination, thereby accelerating pollutant degradation in Au-modified Cu_2_O systems^20^. In addition, recent work on PMS activation with Co_2_VO₄-based catalysts under visible light demonstrated the generation of both ·OH and SO₄·—radicals, leading to nearly complete ciprofloxacin degradation^21^. These findings highlight that alternative oxidant activation strategies, alongside H_2_O_2_, can further broaden the applicability and robustness of catalytic systems for antibiotic degradation.

Unlike previous studies that have focused only on fluoroquinolones, this research examines a wide range of antibiotics and provides diverse degradation pathways. This diversity in antibiotic selection and the examination of their behavior under different environmental conditions complements existing knowledge and contributes to a better understanding of the degradation processes of antibiotic drugs. For example, moxifloxacin is not completely metabolized in living beings and accumulates in wastewater, highlighting the need for robust oxidation strategies to mitigate resistance risks^22^. Likewise, metronidazole, a water-soluble and non-biodegradable antibiotic, is frequently detected in surface waters even at trace concentrations, raising concerns over genotoxicity and resistance gene propagation and underscoring the necessity for efficient degradation methods^10^.

These advances highlight that combining appropriate metals and tailoring active sites are key strategies to enhance catalytic degradation of antibiotics and dyes^12,13^. In this context, the Co_0.5_Fe_0.5_Fe_2_O₄ nanozyme, a magnetic iron–cobalt oxide nanoparticle, offers unique advantages in water treatment applications. The synergistic effect of cobalt and iron enhances redox cycling and provides both catalytic and magnetic properties, while other transition metals such as Cu or Ni, though catalytically active, lack this unique balance^23^. Its robust peroxidase-like activity enables the efficient generation of hydroxyl radicals from hydrogen peroxide under ambient conditions, facilitating the degradation of antibiotics into less harmful intermediates. Moreover, its magnetic properties also allow for easy separation and reuse, making it an environmentally friendly and cost-effective alternative to traditional catalysts^23,24^.

Despite these promising attributes, challenges remain, including potential toxicity, long-term stability, and the need for regeneration of nanozymes for continuous use. Optimizing their synthesis and exploring combinations with other advanced treatment technologies, such as photocatalysis or membrane filtration, could provide synergistic effects, improving pollutant removal and enhancing the degradation efficiency of antibiotics^25,26^.

The Co_0.5_Fe_0.5_Fe_2_O₄ nanozyme has shown significant potential in water treatment applications. Its interdisciplinary applications extend beyond environmental remediation^23^. In hospital settings, it could be employed for the removal of pharmaceutical residues from wastewater, preventing further contamination. Additionally, the nanozyme may have valuable applications in the pharmaceutical industry, where its catalytic properties could be utilized in drug synthesis and wastewater management, addressing the growing challenge of pharmaceutical pollutants.

In this study, the Co_0.5_Fe_0.5_Fe_2_O₄ nanozyme was employed to degrade six widely used antibiotics—ciprofloxacin, azithromycin, levofloxacin, moxifloxacin, amoxicillin, and metronidazole—from aqueous solutions. These antibiotics, selected due to their diverse chemical structures and their prevalence in clinical and veterinary practices, represent different antibiotic classes such as fluoroquinolones, macrolides, beta-lactams, and nitroimidazoles. Their structural complexity, including functional groups like quinolone and nitro rings, contributes to their high resistance to conventional degradation methods, making them persistent environmental pollutants and important targets for this study.

Building on our previous work, where the synthesis, characterization, and removal efficiency of this nanozyme were established under various operational parameters^23^, this research focuses on a detailed investigation of the degradation pathways of these antibiotics using mass spectrometry (MS) to identify intermediate products and elucidate molecular transformations. Furthermore, the extent of mineralization was evaluated through total organic carbon (TOC) analysis to confirm the degradation of the pollutants into less harmful compounds.

**Fig. 1:**
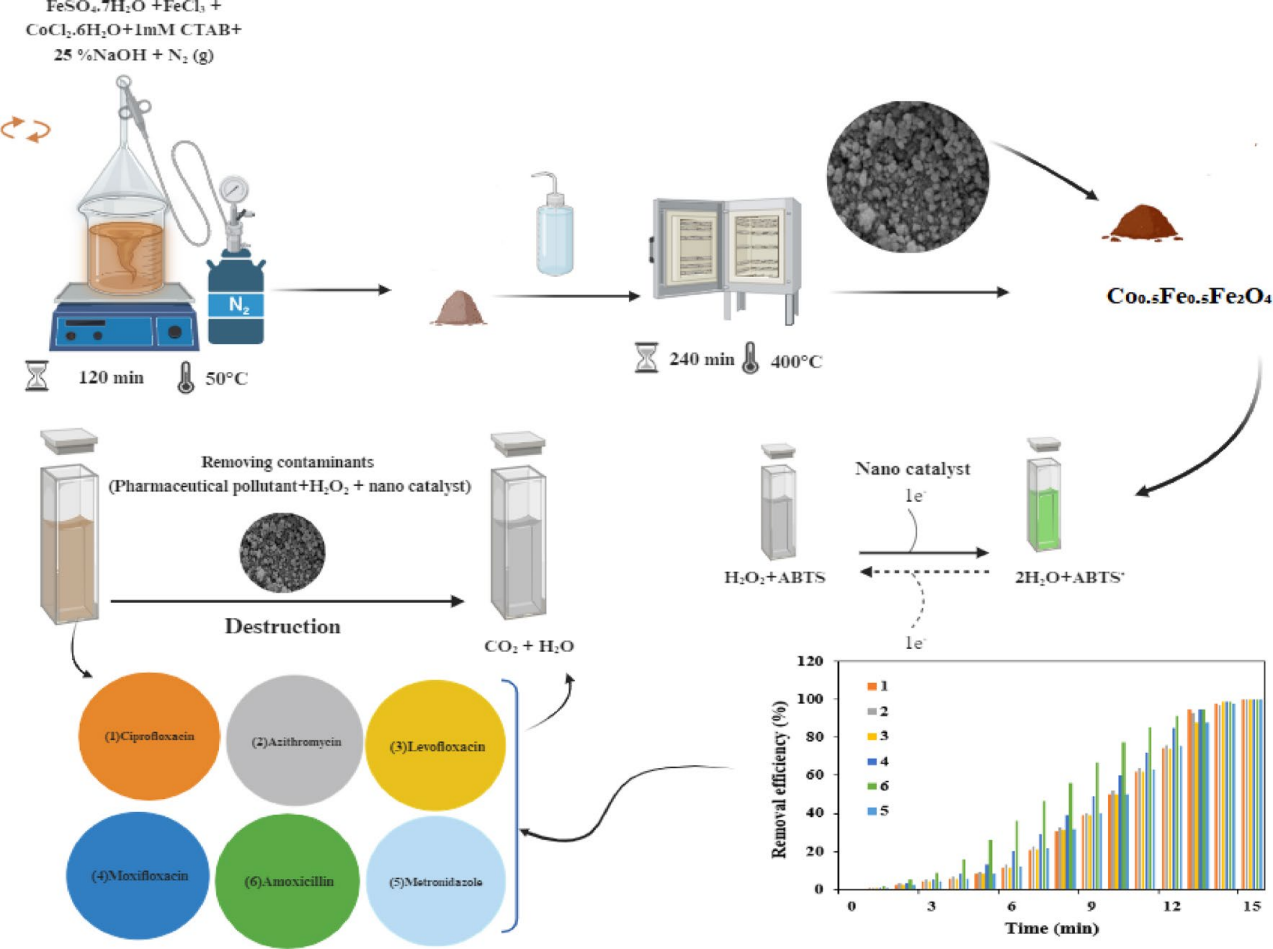
Schematic illustration of Co_0.5_Fe_0.5_Fe_2_O₄ nanozyme and its application for the degradation of six antibiotics (ciprofloxacin, azithromycin, levofloxacin, moxifloxacin, amoxicillin, and metronidazole) through peroxidase-like activity under neutral pH and room-temperature conditions in aqueous solutions.

By integrating these analyses, the research provides deeper insights into the catalytic mechanisms of Co_0.5_Fe_0.5_Fe_2_O₄, assesses its environmental impact, and establishes its feasibility as an advanced and sustainable water treatment solution. The novelty of this study lies in applying a Co_0.5_Fe_0.5_Fe_2_O₄ nanozyme that achieves near-complete degradation of six structurally diverse antibiotics within 15 min at neutral pH and room temperature, conditions where conventional biological, photocatalytic, and Fenton-based processes are often ineffective. In this study, the Co_0.5_Fe_0.5_Fe_2_O₄ nanozyme was employed to degrade six widely used antibiotics—ciprofloxacin, azithromycin, levofloxacin, moxifloxacin, amoxicillin, and metronidazole—from aqueous solutions. Overall, this study aims to elucidate the degradation pathways of six antibiotics and demonstrate the feasibility of Co_0.5_Fe_0.5_Fe_2_O₄ as an efficient and sustainable nanozyme for water treatment. The synthesis process and the catalytic application of the Co_0.5_Fe_0.5_Fe_2_O₄ nanozyme, as previously reported^23^, are illustrated in Fig. 1.

## Materials and methods

### Materials

**Fig. 2:**
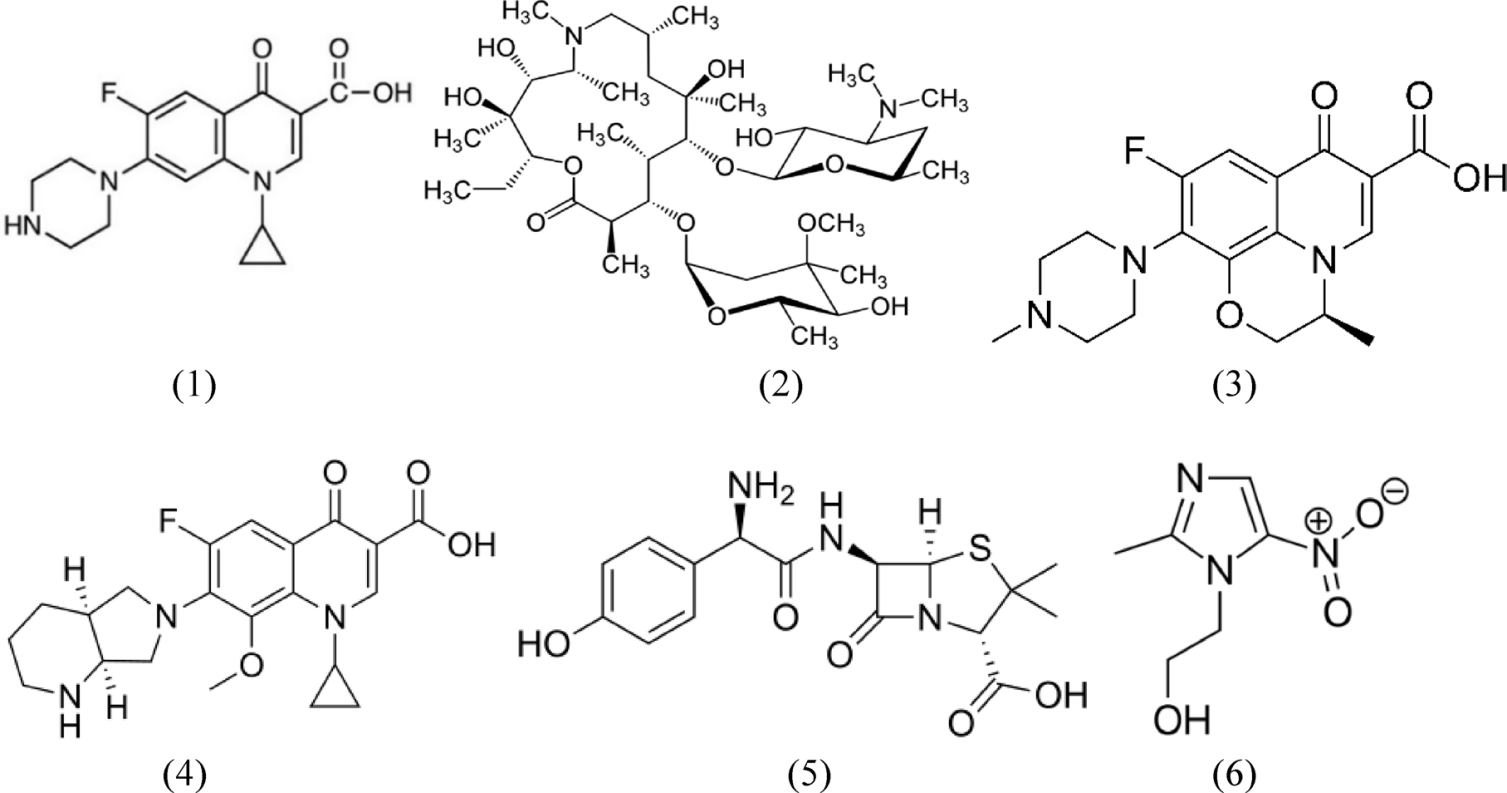
Chemical structures of ciprofloxacin (1), azithromycin (2), levofloxacin (3), moxifloxacin (4), amoxicillin (5), and metronidazole (6).

In this study, six commonly used antibiotics were selected to evaluate the degradation efficiency of the Co_0.5_Fe_0.5_Fe_2_O₄ nanozyme. These antibiotics—ciprofloxacin hydrochloride, azithromycin dihydrate, levofloxacin hemihydrate, moxifloxacin hydrochloride, amoxicillin trihydrate, and metronidazole—represent a diverse range of pharmaceutical compounds commonly found in clinical and environmental contexts. These antibiotics were selected due to their widespread usage in clinical practices and their frequent detection in wastewater, posing significant environmental and health risks. Table 1 summarizes their key characteristics, including molecular formulas, suppliers, maximum absorption wavelengths (λmax), natural pH values, and solubilities in water and purity. Their chemical structures are shown in Fig. 2.

**Table 1:** Characteristics of antibiotics used in the study.

Antibiotic	Chemical formula	Supplier (Country)	Solubility in Water (mg/L)	λ max (nm)	% Purity	Natural pH
Ciprofloxacin	C₁₇H₁₈FN₃O₃.HCl	Zhejiang Guobang Pharmaceutical (China)	30,000	276	≥ 99	~ 6.5
Azithromycin	C₃₈H₇_2_N_2_O₁_2_0.2H_2_O	Century Pharmaceuticals(India)	2500	290	≥ 99	~ 9.5
Levofloxacin	2C₁₈H_2_₀FN₃O₄.H_2_O	Hetero Drugs Limited(India)	25,000	287	≥ 99	~ 6.2
Moxifloxacin	C_2_₁H_2_₄FN₃O₄.HCl	Hetero Drugs Limited(India)	10,000	295	≥ 99	~ 6.0
Amoxicillin	C₁₆H₁₉N₃O₅S.3H_2_O	Fengchen Group Co., Ltd. (China)	4000	229	≥ 99	~ 5.0
Metronidazole	C₆H₉N₃O₃	Hubei Yuanmeng Biological Technology(China)	10,000	277	≥ 99	~ 5.5

Table 1 provides key characteristics of the selected antibiotics, including their natural properties and solubility in water, which are critical for evaluating their degradation efficiency under experimental conditions.

The Co_0.5_Fe_0.5_Fe_2_O₄ nanozyme was synthesized according to the method described in our previous study^23^. Comprehensive synthesis and characterization details—including magnetic properties (VSM), morphology (TEM, SEM), crystal structure (XRD), and elemental composition (EDS) and catalytic properties—are fully provided in the referenced article.In this work, the focus is on elucidating the degradation pathways, where the synthesized nanozyme was employed for the degradation of antibiotics under optimized reaction conditions at neutral pH and room temperature.

### Methods

The degradation experiments of antibiotics using the Co_0.5_Fe_0.5_Fe_2_O₄ nanozyme were performed under optimized conditions reported in our previous study^23^. Antibiotic solutions (0.25 mM) were prepared in deionized water and adjusted to pH 7 using a sodium acetate buffer. Reactions were carried out at room temperature (25 °C) by adding 0.5 mg of the nanozyme together with 0.5 mM hydrogen peroxide (H_2_O_2_) under continuous stirring.

Mineralization efficiency was evaluated by total organic carbon (TOC) analysis (TOC-L CPN, Shimadzu, Japan). Mass spectrometry (LCMS 2010 A, Shimadzu, Japan; ESI mode) was employed to identify intermediate products and elucidate degradation pathways. All analyses were performed in triplicate to ensure reproducibility.

## Results and discussion

### Evaluation and kinetic study of the enzyme-like activity of Co_0.5_Fe_0.5_Fe_2_O₄ nanozyme

The enzyme-like catalytic activity of Co_0.5_Fe_0.5_Fe_2_O₄ was evaluated based on the findings of our previous study^23^ to confirm its peroxidase-mimicking behavior. Using ABTS as a chromogenic substrate in the presence of hydrogen peroxide (H_2_O_2_), the catalytic activity was monitored by the formation of ABTS·⁺, a green-colored product, at 414 nm. The reaction was conducted under optimal conditions: 0.25 mM ABTS, 0.5 mM H_2_O_2_, 0.5 mg nanozyme, and room temperature. The kinetic parameters—Michaelis–Menten constant (*K*_*m*_), maximum velocity (*V*_*max*_), catalytic rate constant (*K*_*cat*_), and catalytic efficiency (*K*_*cat*_/*K*_*m*_)—were determined by fitting the reaction rates to the Michaelis–Menten model^16^. Co_0.5_Fe_0.5_Fe_2_O₄ exhibited a *K*_*m*_ value of 0.0366 mM, reflecting high substrate affinity, and a *V*_*max*_ of 1.10 × 10⁻^4^ mM/s, indicating efficient catalytic performance.The catalytic efficiency (*K*_*cat*_/*K*_*m*_) was calculated as 14.2 mM⁻^1^ s⁻^1^, demonstrating effective peroxidase-like activity under mild environmental conditions.

This exceptional catalytic efficiency can be attributed to the precise co-precipitation synthesis method, which led to the formation of uniform particles with minimal agglomeration, thereby maximizing surface-active sites. By optimizing the stoichiometric ratio of Co (II) and Fe(III), uniformity in the distribution of active sites was achieved, enhancing substrate binding and reaction efficiency. This meticulous synthesis approach ensures a balance that improves the formation of ROS, leading to enhanced degradation rates. A comparative analysis of Co_0.5_Fe_0.5_Fe_2_O₄ with horseradish peroxidase (HRP)^27^ and other nanozymes is summarized in Table 2. While HRP shows higher catalytic turnover rates (*V*_*max*_ = 2.46 × 10⁻^5^ mM/s) and a *K*_*m*_ value of 3.7 mM, its dependence on narrow pH ranges and specific conditions limits its practical applications. Co_0.5_Fe_0.5_Fe_2_O₄, with its high stability and efficiency at neutral pH and ambient temperatures, presents a more viable option for environmental remediation.

**Table 2:** Comparative kinetic parameters of Co_0.5_Fe_0.5_Fe_2_O₄ and other catalysts.

Catalyst	Substrate	*K*_*m*_ (mM)	*V*_*max*_ (mM s⁻^1^)	References
Co_0.5_Fe_0.5_Fe_2_O₄	H_2_O_2_	0.0366	1.10 × 10⁻^4^	This work
HRP	H_2_O_2_	3.7	2.46 × 10⁻5	^27^
MOF(Co/2Fe0)	H_2_O_2_	4.22	0.49 × 10⁻^4^	^28^
Fe_3_O_4_ MNPs	H_2_O_2_	154	9.78 × 10⁻5	^29^
V_2_O_5_	H_2_O_2_	0.232	1.29 × 10⁻2	^29^
GOx-Fe0@EM-A	H_2_O_2_	29.59	3.81 × 10–5	^30^

The comparative analysis of the kinetic parameters, as shown in Table 2, highlights the superior catalytic performance and operational stability of Co_0.5_Fe_0.5_Fe_2_O₄ compared to other synthetic nanozymes, such as MOF(Co/2Fe0)^28^, Fe₃O₄ MNPs, V_2_O₅^29^, and GOx-Fe0@EM-A^30^.Co_0.5_Fe_0.5_Fe_2_O₄ exhibits a remarkably low *K*_*m*_, indicating strong substrate affinity, and competitive *V*_*max*_, underscoring its potential for environmental remediation processes.

From Table 2, it is evident that Co_0.5_Fe_0.5_Fe_2_O₄ exhibits superior substrate affinity, as reflected in its low *K*_*m*_ value, compared to other nanozymes and HRP. Although HRP^27^ has a higher *V*_*max*_, its operational limitations, such as sensitivity to pH and stability issues, restrict its broader applicability. Similarly, Fe₃O₄ MNPs require highly acidic conditions to maintain activity, and MOF (Co/2Fe0) suffers from structural instability in aqueous environments^28,29^. V_2_O₅, on the other hand, requires elevated temperatures for optimal activity, while GOx-Fe0@EM-A depends on complex multi-step synthesis processes, limiting scalability^29,30^. In contrast, Co_0.5_Fe_0.5_Fe_2_O₄ demonstrates enhanced catalytic performance under neutral pH and ambient temperatures—conditions more relevant for practical applications like water treatment. Its ability to operate efficiently in neutral pH without requiring external activation highlights its robustness and suitability for real-world applications^23^.

Another significant advantage of the Co_0.5_Fe_0.5_Fe_2_O₄ nanozyme is its long-term stability. The catalyst maintained nearly 100% of its peroxidase-like activity over a 10-month period, consistent with our previous studies^23^, highlighting its potential for reuse in continuous water treatment processes. Similarly, Ag_2_S/Zn₃V_2_O₈ nanocomposites also exhibited robust stability, where post-reaction XRD and XPS confirmed structural integrity after six consecutive cycles, maintaining > 95% degradation efficiency under visible light irradiation^31^. These findings, together with previous studies on Ag_2_S/Zn₃V_2_O₈ nanocomposites^31^, highlight that transition-metal-based nanozymes can exhibit robust stability under repeated use. In this context, the Co–Fe nanozyme investigated in this work demonstrates similar long-term stability, underscoring its suitability for environmental remediation^23,31^.The redox-active Co(II) and Fe(III) centers in Co_0.5_Fe_0.5_Fe_2_O₄ facilitate efficient generation of reactive oxygen species (ROS), which are critical for degrading pharmaceutical pollutants in aqueous systems. The combination of high catalytic efficiency, stability, and cost-effectiveness positions Co_0.5_Fe_0.5_Fe_2_O₄ as a promising candidate for various environmental and industrial applications.

In addition to peroxidase-like activity, Co_0.5_Fe_0.5_Fe_2_O₄ nanozyme’s potential to integrate with other advanced oxidation processes (AOPs) such as photocatalysis or electrochemical oxidation could further enhance its versatility. By employing a co-precipitation synthesis method and achieving precise control over the stoichiometric balance, this study introduces a nanozyme with superior catalytic properties compared to previously reported systems. Furthermore, it pioneers the application of Co_0.5_Fe_0.5_Fe_2_O₄ in environmental remediation, opening new avenues for its integration with hybrid AOP systems. A comparative assessment indicates that the combination of nanozymes with photo-induced AOPs results in a synergistic effect, improving degradation rates for complex pollutants in challenging environmental conditions. These findings demonstrate the importance of optimizing synthesis conditions, such as controlling precursor concentrations and reaction time, to achieve enhanced stability, efficiency, and durability. Moreover, exploring multi-functional nanozyme composites, such as core–shell structures, may enhance substrate specificity and minimize side reactions. Future studies are required to explore the impact of surface modifications and additional optimization to further enhance the nanozyme’s performance in industrial-scale water treatment applications.

### Removal and degradation mechanisms of antibiotics using Co_0.5_Fe_0.5_Fe_2_O₄ nanozyme

The Co_0.5_Fe_0.5_Fe_2_O₄ nanozyme demonstrated high efficiency in the removal and degradation of six antibiotics—ciprofloxacin, azithromycin, levofloxacin, moxifloxacin, amoxicillin, and metronidazole. Unlike conventional advanced oxidation processes (AOPs), the Co_0.5_Fe_0.5_Fe_2_O₄ nanozyme operates efficiently under neutral pH conditions and does not require UV or light activation. This distinguishes it from commonly used systems that rely on acidic environments or external activation, making it a cost-effective and practical solution for real-world water treatment applications. Leveraging its peroxidase-like activity, the nanozyme catalyzed the decomposition of hydrogen peroxide (H_2_O_2_), generating reactive oxygen species (ROS), such as hydroxyl radicals (·OH) and superoxide anions (O_2_·⁻)^25,28^.

The influence of pH on catalytic performance is closely related to the stability of H_2_O_2_ and the redox cycling of Co/Fe centers. Under acidic conditions, although ·OH radicals are generated efficiently, excessive protonation can destabilize the catalyst surface and accelerate H_2_O_2_ decomposition, lowering overall efficiency. At alkaline pH, H_2_O_2_ undergoes non-radical decomposition to O_2_, leading to reduced ROS generation. In contrast, neutral pH provides an optimal balance, where the Co(II)/Co(III) and Fe(II)/Fe(III) redox pairs remain stable, sustaining efficient ROS production and maximizing antibiotic degradation.

These ROS played a crucial role in degrading the molecular structures of antibiotics, ultimately leading to their mineralization into CO_2_ and H_2_O.

The catalytic process begins with the activation of H_2_O_2_ via redox reactions facilitated by Fe(II) and Co(II) sites on the nanozyme surface:

1 $${\text{H}}_{{2}} {\text{O}}_{{2}} + {\text{Co}}_{{0.{5}}}^{{{2} + }} {\text{Fe}}_{{0.{5}}}^{{{2} + }} {\text{Fe}}_{{2}} {\text{O}}_{{4}} \to^{ \cdot } {\text{OH}} + {\text{OH}}^{ - } + {\text{Co}}_{{0.{5}}}^{{{3} + }} {\text{Fe}}_{{0.{5}}}^{{{3} + }} {\text{Fe}}_{{2}} {\text{O}}_{{4}}$$

**Fig. 3:**
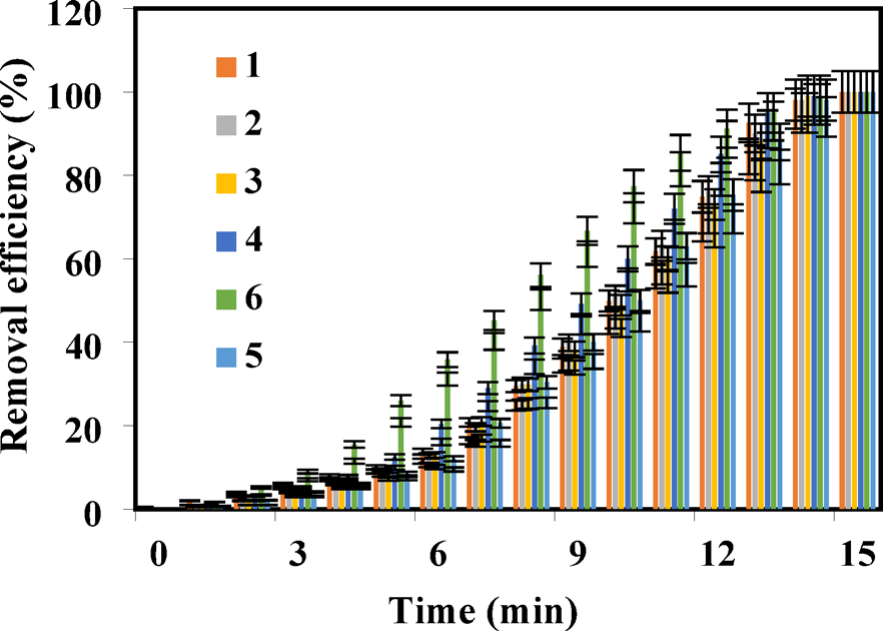
Percentage removal efficiency of antibiotics under optimal conditions: at T = 25 °C and pH = 7, [H_2_O_2_] = 0.5 mM, [Antibiotic] = 0.25 mM, Co_0.5_Fe_0.5_Fe_2_O₄ = 0.5 mg, λmax = (1) 276 nm (ciprofloxacin), (2) 290 nm (azithromycin), (3) 287 nm (levofloxacin), (4) 295 nm (moxifloxacin), (5) 229 nm (amoxicillin), and (6) 277 nm (metronidazole).

**Fig. 4:**
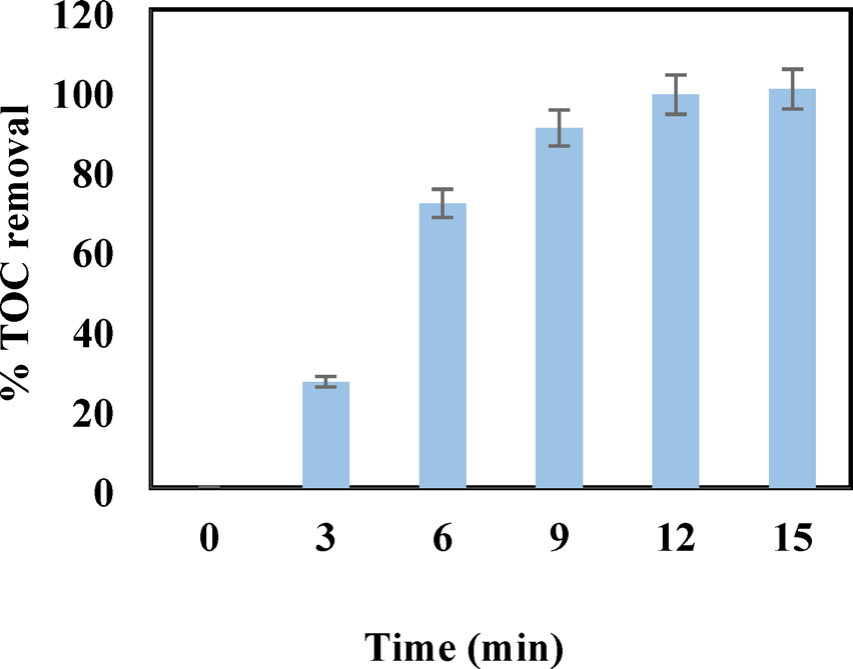
Percentage removal of total organic carbon (TOC) at different time intervals using Co_0.5_Fe_0.5_Fe_2_O₄ Nanozyme under optimal conditions.

In this reaction, the nanozyme’s redox-active sites efficiently decompose H_2_O_2_ to produce ·OH radicals and hydroxide ions, which initiate oxidative degradation. The degradation mechanism of antibiotics in the presence of H_2_O_2_ and Co_0.5_Fe_0.5_Fe_2_O₄ nanozyme involves several oxidative processes primarily driven by the formation of reactive oxygen species (ROS), including hydroxyl radicals (·OH) and superoxide anions (O_2_·⁻). These ROS are generated through the catalytic decomposition of hydrogen peroxide (H_2_O_2_) by the nanozyme. Unlike conventional Fenton reactions that rely heavily on the acidic environment to generate hydroxyl radicals (·OH) efficiently^8,32^, the Co_0.5_Fe_0.5_Fe_2_O₄ nanozyme can operate effectively under neutral pH conditions. This is due to the synergistic effect of the Co(II) and Fe(II) sites, which enhance the redox cycling between Fe(II)/Fe(III) and Co(II)/Co(III) pairs. This dual-metal system promotes more efficient H_2_O_2_ decomposition and ROS generation^8,24^. The generated hydroxyl radicals (·OH) are highly reactive and can attack various functional groups in antibiotic molecules, including amide, hydroxyl, and aromatic groups^33^. This leads to the cleavage of these groups and the subsequent mineralization of the antibiotic molecules into CO_2_, H_2_O, and other harmless by-products. The presence of superoxide anions (O_2_·⁻) further enhances the oxidative degradation process by participating in secondary reactions that regenerate hydrogen peroxide, providing a continuous source of ROS for sustained degradation Sanches-Simões et al.^34–36^. The Co_0.5_Fe_0.5_Fe_2_O₄ nanozyme acts as a peroxidase mimic, where the Fe(II) and Co(II) sites on the nanoparticle surface cyclically undergo redox reactions to catalyze the decomposition of H_2_O_2_ into ·OH radicals. The ROS target key functional groups within antibiotic molecules, cleaving C–C, C–N, and aromatic bonds to generate smaller, less toxic by-products. The removal efficiency of the antibiotics was evaluated under optimized conditions (25 °C, pH 7, 0.5 mM H_2_O_2_, 0.25 mM antibiotic concentration, and 0.5 mg nanozyme). As shown in Fig. 3, near-complete removal was achieved within 15 min. Total organic carbon (TOC) analysis further confirmed the complete mineralization of antibiotics into CO_2_ and H_2_O. Unlike the initial rapid degradation of antibiotic molecules, TOC reduction follows a more gradual trend due to the time required for complete mineralization of intermediate by-products. This behavior aligns with the expected stepwise oxidation mechanism in which complex intermediates degrade progressively into simpler compounds. The TOC results, depicted in Fig. 4, demonstrate that the simultaneous degradation of all six antibiotics was monitored. While the initial degradation of antibiotic molecules occurred rapidly, TOC reduction required slightly more time due to the sequential nature of the process—initial molecular degradation followed by full mineralization into simpler compounds. Fluoroquinolones such as ciprofloxacin, levofloxacin, and moxifloxacin showed high removal efficiencies due to their reactive piperazine and quinolone cores, which are highly susceptible to hydroxyl radical attacks. Amoxicillin’s β-lactam ring facilitated rapid degradation through oxidation and ring cleavage. Despite its complex macrolide structure, Azithromycin was effectively degraded, demonstrating the broad substrate applicability of the nanozyme. Metronidazole’s nitro group (-NO_2_) and imidazole ring provided reactive sites for hydroxyl radicals, leading to efficient degradation involving nitro group cleavage and imidazole ring destruction. Detailed MS-derived degradation pathways for each antibiotic are summarized in Table 3, with full fragmentation data provided in the Supplementary Information (Figures S1–S6 and Tables S1–S6).The degradation mechanisms involved several oxidative steps driven by ROS, particularly hydroxyl radicals (·OH) and superoxide anions (O_2_·⁻). These ROS degraded functional groups within the antibiotic molecules, including amide, hydroxyl, and aromatic groups. The process culminated in the degradation of these groups, yielding smaller by-products such as carboxylic acids, aldehydes, and finally CO_2_ and H_2_O. Superoxide anions (O_2_·⁻) further enhanced the process by participating in secondary reactions that regenerated hydrogen peroxide, sustaining ROS production. Mass spectrometry (MS) analysis provided detailed information about the intermediates formed during the degradation of each antibiotic, helping to further elucidate the pathways and key functional group transformations. The observed shifts in the mass-to-charge (m/z) ratios over time were consistent with the proposed degradation steps, confirming the sequential degradation of the antibiotic molecules into simpler and safer products^9,32–34,37–40^. These insights reinforce the efficiency and specificity of the Co_0.5_Fe_0.5_Fe_2_O₄ nanozyme in environmental remediation applications.

**Table 3:** Degradation pathways, key intermediates, and toxicity of antibiotics using Co_0.5_Fe_0.5_Fe_2_O₄ nanozyme.

Antibiotic	Structure targeted	Degradation pathway	Key intermediates	Final products	Notes on toxicity
Ciprofloxacin	Piperazine ring, aromatic quinolone core	Hydroxylation, decarboxylation, oxidation/opening of piperazine ring, cleavage of the quinolone core, Followed by deep oxidation ,mineralization	Carboxylic acids, aldehydes, (trace intermediates)	CO_2_, H_2_O	Toxicity of by-products needs further study
Azithromycin	Lactone ring, methylene (CH_2_) groups, nitrogen-containing moieties	O/N-demethylation, hydroxyl radical attack, ring opening, side-chain oxidation, deep fragmentation, mineralization	Aldehydes, ketones, carboxylic acids, (trace intermediates)	CO_2_, H_2_O	Exact toxicity of by-products is undetermined
Levofloxacin	Quinolone ring system, piperazine moiety	Hydroxylation/oxidation, decarboxylation, ring cleavage, piperazine ring opening, deep fragmentation, mineralization	Amides, aldehydes, aromatic/aliphatic fragments, (trace intermediates)	CO_2_, H_2_O	Environmental toxicity impact should be further evaluated
Moxifloxacin	uinolone ring, methoxy group	Oxidation / demethoxylation oxidation/ decarboxylation, ring cleavage/partial ring opening,deep fragmentation, mineralization	Aliphatic and aromatic intermediates, carboxylic acids, (trace intermediates)	CO_2_, H_2_O	Comprehensive toxicological evaluations needed
Amoxicillin	Beta-lactam ring, thiazolidine ring, amino group	β-lactam cleavage, S-oxidation, deamination, thiazolidine oxidation, side-chain fragmentation, deep oxidative fragmentation, mineralization	Penicilloic acid, aldehydes, (trace intermediates; sulfate tentative in ESI)	CO_2_, H_2_O	Environmental impact and toxicity of by-products require study
Metronidazole	Nitro group (-NO_2_), imidazole ring	N–O bond scission, nitro reduction, oxidative cleavage of imidazole, deep oxidative fragmentation, mineralization	Nitroso (R-NO), hydroxylamine (R-NHOH), (trace intermediates; nitrate/nitrite tentative in ESI)	CO_2_, H_2_O	Further studies needed to conform toxicity of nitrogenous by-product

The degradation pathways presented in Table 3, elucidated through mass spectrometry (MS) analysis, reveal differences and specific structural vulnerabilities in each antibiotic, which influence their degradation into less complex products. These pathways provide valuable insights for optimizing degradation conditions and minimizing the formation of toxic by-products in environmental applications .Compared to conventional advanced oxidation processes (AOPs), the Co_0.5_Fe_0.5_Fe_2_O₄ nanozyme demonstrated superior versatility and efficiency. While Fe₃O₄ nanoparticles are effective under acidic conditions, their activity significantly decreases at neutral pH. Photocatalysts such as TiO_2_ and ZnO require light activation, complicating operational conditions and increasing costs. Fenton-based processes, although highly efficient, are limited by stringent pH requirements and generate large amounts of sludge [9, 16, and 41]. In contrast, the Co_0.5_Fe_0.5_Fe_2_O₄ nanozyme operates efficiently at neutral pH and ambient temperature, providing a cost-effective and practical solution for water treatment applications. Its ability to degrade structurally diverse antibiotics and mineralize them into non-toxic by-products highlights its environmental significance. Despite the effective degradation of various antibiotics, the formation of by-products such as nitrite and nitrate ions during metronidazole degradation highlights the need for further toxicological evaluations. Additionally, the variability in degradation efficiency depending on functional groups emphasizes the importance of optimizing nanozyme reactivity for diverse antibiotic structures. By degrading antibiotics into simpler and safer products, the Co_0.5_Fe_0.5_Fe_2_O₄ nanozyme demonstrated exceptional potential for environmental remediation. These findings highlight the significant potential of Co_0.5_Fe_0.5_Fe_2_O₄ nanozyme for environmental remediation, particularly in antibiotic-contaminated waters. However, further investigations into the toxicity of degradation by-products, optimization of surface properties, and performance evaluations in large-scale settings are crucial to ensure both efficacy and environmental safety.

The degradation of different antibiotic classes exhibited significant variations in terms of both the pathway and efficiency^8,24^. Fluoroquinolones, such as ciprofloxacin, levofloxacin, and moxifloxacin, were rapidly degraded due to their reactive cores, while antibiotics like Amoxicillin and Azithromycin, which have more complex structures, showed slower degradation rates. The presence of functional groups like the nitro group in Metronidazole and the β-lactam ring in Amoxicillin also influenced the degradation efficiency, contributing to the formation of various intermediates over time. These variations in degradation dynamics are essential for optimizing nanozyme performance in diverse environmental contexts, highlighting the need for tailored treatment approaches based on antibiotic structure.

### Comparative assessment and environmental implications of Co_0.5_Fe_0.5_Fe_2_O₄ nanozyme performance

The catalytic performance of the Co_0.5_Fe_0.5_Fe_2_O₄ nanozyme was evaluated based on its ability to degrade various antibiotics under oxidative conditions. As shown in Fig. 3, a remarkable increase in the removal efficiency of contaminants over time highlights the exceptional potential of Co_0.5_Fe_0.5_Fe_2_O₄ in oxidative degradation processes. Unlike conventional photocatalysts that rely on continuous light exposure, Co_0.5_Fe_0.5_Fe_2_O₄ operates efficiently at neutral pH and ambient temperature, showcasing its suitability for real-world applications.

Several studies have documented the use of alternative nanocatalysts for antibiotic degradation. For example,^24^ demonstrated that Co-substituted Fe₁₋ₓS (CSP) achieved ~ 97% tetracycline removal within 40 min using a heterogeneous Fenton process. This efficiency was attributed to enhanced Fe^2^⁺/Fe^3^⁺ redox cycling, but the optimal pH of 5 limits its application under neutral conditions. Similarly,^42^ reported ~ 95% and ~ 90% removal of tetracycline hydrochloride and doxycycline, respectively, using a biochar-supported nano manganese dioxide composite (BC/MnO_2_) via an adsorption-based mechanism. However, the adsorption process required 420 min to reach significant removal, highlighting its slower kinetics compared to Co_0.5_Fe_0.5_Fe_2_O₄.

In contrast, Co_0.5_Fe_0.5_Fe_2_O₄ achieves near-complete removal of multiple antibiotics (ciprofloxacin, azithromycin, levofloxacin, moxifloxacin, amoxicillin, and metronidazole) within just 15 min. This rapid degradation occurs under neutral pH, with a low concentration of hydrogen peroxide as the oxidant ([H_2_O_2_] = 0.5 mM), highlighting its efficiency,^23^. Unlike ZnO/Cu_2_O/g-C₃N₄ heterojunctions, which exhibited 98.79% tetracycline degradation in 30 min under visible light,^35^, and CuO/ZnO heterojunction photocatalysts, which degraded ciprofloxacin (93%) and tetracycline (94%) in 50 min under sunlight Bano,^43^, Co_0.5_Fe_0.5_Fe_2__2_O₄ does not rely on external light sources. This independence from UV or visible light makes it more energy-efficient and scalable for wastewater treatment applications.

The dual-metal composition of Co_0.5_Fe_0.5_Fe_2_O₄ is a critical factor enhancing its catalytic efficiency. The synergistic redox cycling between Co(II)/Co(III) and pairs leads to increased reactive oxygen species (ROS) generation,^24,28^. Single metal oxides like Fe₃O₄, in contrast, often show reduced ROS yields and lower efficiency under neutral pH conditions,^8,32^. Furthermore, the chemical structure of certain antibiotics, such as the β-lactam ring in amoxicillin and the nitro group in metronidazole, renders them particularly susceptible to ROS attacks,^32,34^. This structural vulnerability, coupled with the high ROS yield of Co_0.5_Fe_0.5_Fe_2_O₄, contributes to its superior performance.

Compared to adsorption-based systems like Fe₃O₄-ACLM, which achieved 100% ciprofloxacin removal in 75 min,^44^, Co_0.5_Fe_0.5_Fe_2_O₄ offers a faster, catalytic alternative without concerns about surface saturation. Another example is MWCNTs-CuNiFe_2_O₄, which achieved 100% amoxicillin degradation within 80–120 min through peroxymonosulfate (PMS) activation,^32^. However, this system required a high catalyst dosage of 500 mg/L, significantly increasing material costs. In contrast, Co_0.5_Fe_0.5_Fe_2_O₄ achieved superior results with only 0.5 mg of catalyst, making it more cost-effective. The comparative analysis presented in Table 4 highlights the superior adaptability and rapid action of Co_0.5_Fe_0.5_Fe_2_O₄ under diverse conditions. Unlike photocatalysts like g-C₃N₄/La–N-TiO_2_ composites, which rely on visible light (96.8% ciprofloxacin degradation in 60 min),^45^, Co_0.5_Fe_0.5_Fe_2_O₄ functions under ambient light, reducing energy demands.

**Table 4:** Comparative overview of nanocatalysts used for antibiotic degradation in aqueous solutions.

Study	Nanocatalyst	Antibiotics targeted	Removal efficiency (%)	Time (min)	Conditions
This study	Co_0.5_Fe_0.5_Fe_2_O₄ nanozyme	Ciprofloxacin, Azithromycin, Levofloxacin, Moxifloxacin, Amoxicillin, Metronidazole	~ 100.00	15	Peroxidase-like catalytic degradation with hydrogen peroxide (H_2_O_2_). [Antibiotic] = 0.25 mM, [H_2_O_2_] = 0.5 mM , Nanozyme = 0.5 mg, pH = 7, T = 25 °C
Wang et al.^24^	Co-substituted Fe₁₋ₓS (CSP)	Tetracycline	~ 97.00	40	Heterogeneous Fenton process; catalyst Dose = 0.1 g/L, [H_2_O_2_] = 100 µM. PH = Optimal at 5.0, effective range 3.0–9.0 T = 25 °C
Rahmani et al.^32^	MWCNTs-CuNiFe_2_O₄	Amoxicillin	100.00	80–120	Peroxymonosulfate (PMS) activativation; catalyst dosage = 500 mg/L Peroxymonosulfate (PMS) dosage = 5 mM, [AMX ] = 50 mg/L, pH = 7 T = 25 °C
Sanches-Simões et al.^34^	TiO_2_ nanoparticles functionalized on a polyurethane macroporous structure (TiO_2_@PU)	Metronidazole	> 97.00	10	A combination of advanced oxidative processes (AOPs) leveraging physical (US, UV) and chemical (H_2_O_2_, O₃) + TiO_2_ nanoparticles [MNZ] = 20 mg/L [H_2_O_2_] = 8 mM [O₃] = 20 mg/L, pH = 7, T = 25 °C
Zhu et al.^35^	ZnO/Cu_2_O/g-C₃N₄ heterojunction	Tetracycline Chlortetracycline Oxytetracycline Ciprofloxacin	98.79 99.50 95.35 73.53	30	Visible-light photocatalysis Light Source: Visible light (λ > 420 nm) pH = alkaline favored, [Catalyst] = 0.5 g/L
Li et al.^42^	Biochar-supported nano manganese dioxide composite (BC/MnO_2_)	Tetracycline hydrochloride doxycycline	~ 95.00 ~ 90.00	420	Adsorption , [Antibiotic ] = 5–100 mg/L, Adsorbent dose = 0.1 g /25 mL pH = 6, T = 25 °C
Bano et al.^43^	CuO/ZnO heterojunction photocatalyst	Tetracycline Ciprofloxacin	94.00 93.00	50	Sunlight driven photocatalysis Catalyst Dosage = 30 mg, [Antibiotic] = 25 mg/L, pH = 6
Yilmaz et al.^44^	Fe₃O₄-ACLM (activated carbon derived from Lemna minor plant, magnetized with Fe₃O₄ nanoparticles)	Ciprofloxacin	100.00	75	Adsorption , [CIP ] = 25 mg/L Nanoparticle dosage = 0.75 g/L, pH = 3
Yu et al.^45^	g-C3N4/La–N-TiO2 composite	Ciprofloxacin	96.80	60	Visible-light photocatalysis, [CIP] = 10 mg/L Catalyst dosage = 0.75 g/L Light source = Simulated solar light with a 300 W Xe lamp (λ > 420 nm), pH = Approximately 6.5 (optimal range 6–7)
Subhiksha et al.^46^	γ-Bi_2_O₃/CoFe_2_O₄/Ag photocatalyst	Ciprofloxacin	96.60	220	Visible-light photocatalysis, [CIP]. = 10 mg/L; Catalyst dosage = 1 mg/L; pH ≈ 6; λ > 420 nm
Nazeer Ali et al.^47^	BiOCl/LaNi₄Fe nanohybrid	Levofloxacin	96.30	150	Visible-light photocatalysis, Catalyst dosage = 200 mg in 20 mL solution (10 mg/L LEV) with H_2_O_2_; pH optimum = 3; T = 25 °C,
Sabariselvan et al^48^	Sulfur-defect engineered ZnS (S-dZ, 1:1 ratio)	Levofloxacin	~ 88.00% (TOC = ~ 86.80%)	120	Visible-light photocatalysis, degradation under 1000 W halogen lamp (visible light, 75,000 lx),, ,Catalyst dose = 100 mg/L [ LVO] = 20 mg/L, pH:7
Sruthi et al.^49^	LaVO₄/Bi₁_2_O₁₇Br_2_ nanocomposite	Metronidazole	98.30	90	Visible-light photocatalysis, (λ > 400 nm, 500 W tungsten lamp); [MET] = 20 mg/L, Catalyst dosage = 75 mg/L, pH = 4; pseudo-first-order kinetics; ·OH and h⁺ as main reactive species
Vinotha Sre^50^	Ag@ZnCdS QDs Schottky heterojunction	Metronidazole	~ 95.00	260	Visible-light photocatalysis, Catalyst dose = 50 mg/L (optimized between 25–75 mg/L), [MNZ]20 mg/L (tested range 10–50 mg/L, efficiency decreases slightly at higher concentrations) , pH: Neutral to alkaline (pH 4–9, efficiency stable ~ 95%, but drops at pH 3 to 87%) ,Light source: Visible light (1000 W halogen lamp),Added oxidant: H_2_O_2_ (500 μL) to enhance degradation,
Rezaei Kalantary, et al.^52^	Nano-Fe₃O₄ (magnetite nanoparticles)	Amoxicillin	98.20	60	Electro-Fenton process, pH: Highly acidic conditions Catalyst Dosage = 1 g/L, [Amoxicillin ] = 20 mg/L pH = 3, Applied Current = 300 mA, Distance Between Electrodes: 1 cm
Zulfiqar, et al.^53^	Magnetic nanocomposite (Fe3O4 biochar)	Ciprofloxacin Amoxicillin	73.51 74.07	240	UV photocatalysis , Ciprofloxacin : pH = 6, Catalyst Dosage = 0.12 g [Ciprofloxacin] = 100 mg/L Amoxicillin: pH = 5, Catalyst Dosage = 0.12 g, [Amoxicillin] = 100 mg/L
Shi et al.^54^	Ag/PW12/TiO2 composite (10% Ag/PT is the optimized catalyst)	Tetracycline Enrofloxacin	78.19 93.65	60	Visible-light photocatalysis, Catalyst Dosage = 20 mg, pH: 7 for TC and 11 for optimal degradation of ENR [Antibiotic] = 20 ppm (20 mL solution) Light Source: 300 W Xenon lamp (λ > 420 nm)
Apostolescu et al.^55^	CeO_2_/ZnO heterojunctions	Chlortetracycline Ceftriaxone	71.23 58.65	120	UV photocatalysis, [Chlortetracycline ] = 0.025 g·L⁻^1^, [Ceftriaxone] = 0.05 g·L⁻^1^ , Catalyst dose = 0.05 g·L⁻^1^ , pH = 6, T = 25 °C, UV Source: UV-B lamp (OSRAM, 18 W), 0.21 mW·cm⁻^2^ radiant flux
Bai et al.^56^	Magnetic nanocomposite based on Fe3O4 combined with graphene oxide	Amoxicillin Ciprofloxacin	90.88 93.48	240	Visible-light photocatalysis, pH = Ciprofloxacin: 6, Amoxicillin: 5 Catalyst Dose = 0.12 g/L [Antibiotic] = 100 mg/L

In addition, recent studies^46–50^ have also reported efficient degradation of various antibiotics using novel nanocatalysts, further supporting the importance of transition-metal-based systems for pharmaceutical wastewater treatment.

The comparative data summarized in Table 4 illustrate the removal efficiencies, conditions, and operating methodologies for different nanocatalysts, showcasing Co_0.5_Fe_0.5_Fe_2_O₄ as a robust and versatile candidate for environmental remediation, especially in antibiotic-contaminated waters^24,32,34,35,42–56^.

These findings underscore the exceptional potential of Co_0.5_Fe_0.5_Fe_2_O₄ as an efficient, scalable, and sustainable nanocatalyst for environmental remediation, particularly in antibiotic-contaminated waters. Future studies could focus on hybridizing Co_0.5_Fe_0.5_Fe_2_O₄ with advanced oxidation processes, such as photo-Fenton-like reactions, to further enhance its degradation capabilities and long-term stability.

### Environmental and safety considerations of Co_0.5_Fe_0.5_Fe_2_O₄ nanozyme

It is important to address potential environmental and safety concerns associated with the use of Co_0.5_Fe_0.5_Fe_2_O₄ nanozymes. The persistence of nanozyme particles in natural water bodies and the formation of potentially toxic degradation by-products require careful examination. Long-term studies, as suggested by^41,57^, are needed to evaluate the safety of nanozymes in environmental applications and to ensure that their use does not introduce new environmental risks. Future research should prioritize understanding the fate and transport of nanozyme particles in real-world environments and evaluating the biocompatibility and as well as toxicity of degradation by-products.

The kinetic stability analysis provides deeper insights into the complex mechanisms involved in antibiotic removal^23^. The presence of contaminants introduces a competitive environment for the active sites of the catalyst during the oxidation process. Despite this competition, the strong interaction between the antibiotics and the catalyst surfaces may lead to catalyst deactivation, reducing the availability of active sites. Nanoparticle aggregation also plays a significant role in limiting the oxidation process. The kinetic analysis findings substantiate the proposed inhibitory mechanism, confirming that antibiotic pollutants block the catalytic sites of Co_0.5_Fe_0.5_Fe_2_O₄, which negatively impacts its oxidation capacity. These findings suggest that although Co_0.5_Fe_0.5_Fe_2_O₄ exhibits excellent catalytic properties, challenges such as active site inhibition and nanoparticle aggregation must be addressed to optimize its performance^23^. In light of these challenges, future research should explore surface modifications or stabilizing agents to prevent aggregation and maintain high catalytic efficiency. Additionally, it is crucial to understand how different water chemistries and the presence of other pollutants influence Co_0.5_Fe_0.5_Fe_2_O₄’s performance. The long-term environmental impacts of using such nanozymes in aquatic systems also require careful evaluation. Although Co_0.5_Fe_0.5_Fe_2_O₄ nanozymes have demonstrated high efficiency in degrading antibiotics, the stability and persistence of the degradation by-products require further examination. Incomplete degradation or the formation of secondary pollutants could pose additional ecological risks. These by-products might retain biological activity or exert toxic effects, necessitating comprehensive toxicological evaluations to ensure their safe release into the environment^5,17^. Compared to traditional methods and other nanocatalysts, Co_0.5_Fe_0.5_Fe_2_O₄ stands out as a robust and efficient option for pharmaceutical pollutant remediation. However, mitigating the effects of aggregation and optimizing conditions are essential to maintain high degradation efficiency in varied environmental contexts. Moreover, understanding the fate and interactions of nanozyme particles in natural water bodies is crucial for evaluating their broader environmental safety. Pilot-scale studies under real wastewater treatment conditions are essential to assess the potential leakage of cobalt and iron ions and the long-term interactions of the nanozyme with the environment. By addressing these aspects, Co_0.5_Fe_0.5_Fe_2_O₄ can become a key component in sustainable water treatment solutions.

## Conclusion

The Co_0.5_Fe_0.5_Fe_2_O₄ nanozyme demonstrates remarkable potential as an efficient catalyst for the degradation of various antibiotics in aqueous solutions. Its superior catalytic performance, driven by peroxidase-like activity, enables the generation of reactive oxygen species (ROS), such as hydroxyl radicals (·OH) from hydrogen peroxide (H_2_O_2_). In this study, the nanozyme achieved near-complete removal of six representative antibiotics (ciprofloxacin, azithromycin, levofloxacin, moxifloxacin, amoxicillin, and metronidazole) within 15 min under neutral pH and room-temperature conditions. TOC analysis confirmed substantial mineralization, while MS analysis identified the key degradation intermediates and elucidated distinct pathways for each antibiotic. While many previous studies have focused on photocatalysts or Fenton-like systems that require acidic conditions or external irradiation, the present work addresses a critical research gap by demonstrating efficient degradation of structurally diverse antibiotics under neutral pH and ambient conditions. Moreover, the mechanistic insights obtained from MS and TOC analyses extend beyond conventional approaches, providing a comprehensive understanding of antibiotic degradation pathways. However, operational challenges, such as competitive interactions at active sites and nanoparticle aggregation, may reduce catalytic efficiency. To address these challenges, future research should focus on improving surface properties to prevent aggregation and maintain the availability of active sites. Additionally, further studies under complex water matrices and in the presence of co-existing pollutants will provide insights into its real-world performance. The potential of Co_0.5_Fe_0.5_Fe_2_O₄ for integration into wastewater treatment systems is particularly noteworthy due to its high degradation efficiency, stability, and reusability. For large-scale implementation, optimizing the synthesis process to ensure cost-effectiveness and scalability is essential. This includes refining particle size, surface area, and active site distribution to maximize catalytic performance. Combining Co_0.5_Fe_0.5_Fe_2_O₄ with other advanced oxidation processes (AOPs) or photocatalytic systems could further enhance its efficiency and broaden its applications. Moreover, it is crucial to investigate the potential formation of degradation by-products during antibiotic degradation. Although most intermediates identified in this work were further oxidized into CO_2_ and H_2_O, additional toxicological evaluations are recommended. In conclusion, Co_0.5_Fe_0.5_Fe_2_O₄ presents a promising, practical, and sustainable solution for degrading antibiotics and other organic contaminants in water. Its robustness, high catalytic activity, and ease of recovery underscore its potential for environmental and pharmaceutical waste management. Nonetheless, continued research is essential to address current limitations, optimize operational parameters, and expand its use across various environmental and industrial contexts. The detailed mechanistic insights obtained here can guide the development of safer water purification technologies and contribute to protecting ecosystems from pharmaceutical pollutants. Overall, this study uniquely demonstrates that a reusable Co–Fe nanozyme can efficiently degrade structurally diverse antibiotics under environmentally relevant mild conditions, providing mechanistic insights that are not available from conventional methods.

## Supplementary Information

Supplementary Information.

## Data Availability

The data that support the findings of this study are available from the corresponding author upon reasonable request.
