# Supplementary material for: Exploration of degradation pathways of six antibiotics using a novel Co0.5Fe0.5Fe2O₄ nanozyme
Source: Sci Rep. 2025 Nov 14;15:39941. doi: 10.1038/s41598-025-23740-2 (PMC12618689; doi:10.1038/s41598-025-23740-2)
Supplement: Supplementary file 1 — Supplementary Information. [file 41598_2025_23740_MOESM1_ESM.docx]

**
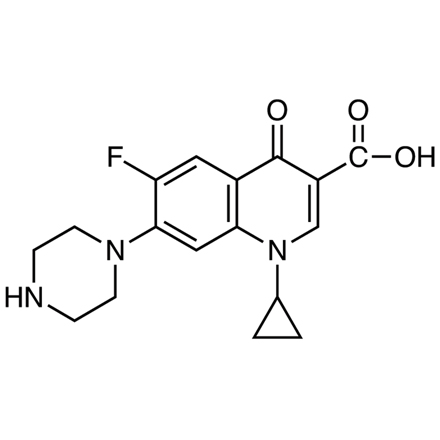
**

Main pathway:

CIP-1 (m/z 332.13)

↓ (hydroxylation, oxidation of piperazine)

CIP-3 (m/z 304.13) / CIP-4 (m/z 304.11, positional isomer)

↓ (decarboxylation)

CIP-2 (m/z 282.10, transient) → CIP-5 (m/z 268.11)

↓ (ring cleavage)

CIP-6 (187.05), CIP-7 (m/z 105.07) / CIP-8 (m/z 77.04, trace fragment)

↓ (further oxidation → mineralization)

CO₂ + H₂O

**Fig. S1.** Degradation pathway of ciprofloxacin under Co₀.₅Fe₀.₅Fe₂O₄/H₂O₂ system

**Table S1.** Detected intermediates of ciprofloxacin degradation at different reaction times under Co₀.₅Fe₀.₅Fe₂O₄/H₂O₂ system (MS analysis).

| **Time** | **Intermediates (m/z)** |
| --- | --- |
| 5 min | CIP-1 (332.13), CIP-2 (282.10) |
| 9 min | CIP-2 (282.10), CIP-3 (304.13), CIP-4 (304.11) |
| 12 min | CIP-3 (304.13), CIP-5 (268.11), CIP-7 (105.07) CIP-6 (187.05), CIP-8 (77.04) |
| 15 min | CO₂ (44), H₂O (18); trace intermediates: CIP-7 )105.07(,CIP-8 )77.04( |

Note: trace = very low-intensity MS signal (≤5% relative to the base peak intensity); ND = not detected above the instrument detection limit.

Note: Sulfate, nitrate, and nitrite ions were only tentatively detected due to the limited sensitivity of ESI-MS for inorganic anions.


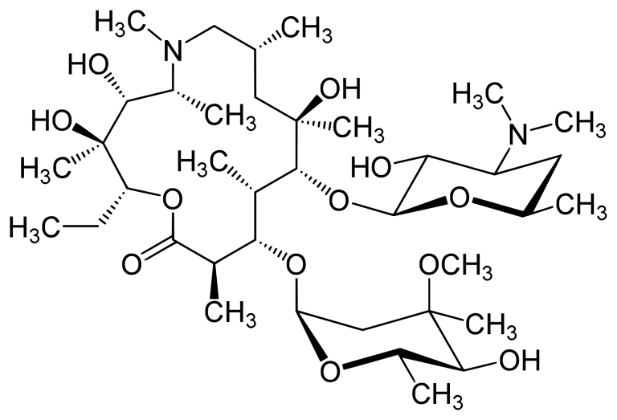


Main pathway:

AZI-1 (m/z 748.51, parent)

↓ (O/N-demethylation)

AZI-2 / AZI-4 (m/z 734.49, positional isomers)

↓ (demethylation / ring opening / oxidation–hydrolysis)

AZI-3 (m/z 716.45)

↓ (oxidation)

AZI-5 (m/z 720.48)

↓ (fragmentation / lactone opening)

AZI-6 (m/z 702.43)

↓ (deep fragmentation)

AZI-7 (m/z 591.40)

↓ (further cleavage, minor branch)

AZI-8 (m/z 433.30)

↓ (final oxidation)

CO₂ + H₂O

**Fig. S2.** Degradation pathway of azithromycin under Co₀.₅Fe₀.₅Fe₂O₄/H₂O₂ system

**Table S2.** Detected intermediates of azithromycin degradation at different reaction times under Co₀.₅Fe₀.₅Fe₂O₄/H₂O₂ system (MS analysis).

| **Time** | **Intermediates (m/z)** |
| --- | --- |
| 5 min | AZI -1 (748.5085), AZI -2 (734.4928) |
| 9 min | AZI -2 (734.4928), AZI -3 (716.4459),AZI-4 (720.4772), |
| 12 min | AZI-4 (720.4772), AZI -5 (702.4302) AZI -6 (187.05), AZI-7 (591.3982),AZI-8(433.3039) |
| 15 min | CO₂ (44), H₂O (18); trace intermediates: AZI-7 (591.3982), AZI-8 (433.3039) |

Note: trace = very low-intensity MS signal (≤5% relative to the base peak intensity); ND = not detected above the instrument detection limit.

Note: Sulfate, nitrate, and nitrite ions were only tentatively detected due to the limited sensitivity of ESI-MS for inorganic anions.


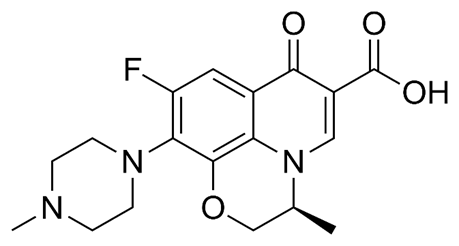


Main pathway :

LFX-1 (m/z 362) (parent)

↓ (oxidation / hydroxylation)

LFX-2 (m/z 323)

↓ (decarboxylation)

LFX-3 (m/z 318)

↓ (ring opening / cleavage)

LFX-4 (m/z 290), LFX-5 (m/z 279)

↓ (deep fragmentation)

LFX-6 (m/z 215) → LFX-9 (m/z 101) / LFX-7 (m/z 115) / LFX-8 (m/z 61)

↓ (further oxidation → mineralization)

CO₂ + H₂O

**Fig. S3.** Degradation pathway of levofloxacin under Co₀.₅Fe₀.₅Fe₂O₄/H₂O₂ system

**Table S3.** Detected intermediates of levofloxacin degradation at different reaction times under Co₀.₅Fe₀.₅Fe₂O₄/H₂O₂ system (MS analysis).

| Time | Detected species (m/z) |
| --- | --- |
| 5 min | LFX-1 (362), LFX-2 (323) |
| 9 min | LFX-2 (323), LFX-3 (318) |
| 12 min | LFX-3 (318), LFX-4 (290), LFX-5 (279), LFX-6 (215); small fragments: LFX-7 (115), LFX-8 (61), LFX-9 (101) |
| 15 min | CO₂ (44), H₂O (18); trace intermediates: LFX-9 (101), LFX-8 ( 61 ) |

Note: trace = very low-intensity MS signal (≤5% relative to the base peak intensity); ND = not detected above the instrument detection limit.

Note: Sulfate, nitrate, and nitrite ions were only tentatively detected due to the limited sensitivity of ESI-MS for inorganic anions.


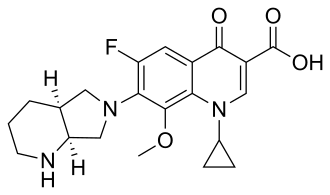


Main pathway :

MFX-1 (m/z 402)

↓ (oxidation / demethoxylation)

MFX-2 (m/z 384)

↓ (oxidation / decarboxylation)

MFX-3 (m/z 358)

↓ (ring cleavage / oxidation)

MFX-4 (m/z 330), MFX-5 (m/z 302)

↓ )further oxidation / partial ring opening(

MFX-6 (m/z 286)

↓ )deep fragmentation & oxidative cleavage

MFX-7 (m/z 258), MFX-8 (m/z 230), MFX-9 (m/z 202)

↓ (further oxidation → mineralization)

CO₂ + H₂O

**Fig. S4.**Degradation pathway of moxifloxacin under Co₀.₅Fe₀.₅Fe₂O₄/H₂O₂ system

**Table S4.** Detected intermediates of moxifloxacin degradation at different reaction times under Co₀.₅Fe₀.₅Fe₂O₄/H₂O₂ system (MS analysis).

| **Time** | **Intermediates (m/z)** |
| --- | --- |
| 5 min | MFX-1 (402), MFX-2 (384) |
| 9 min | MFX-2 (384), MFX-3 (358) |
| 12 min | MFX-3 (358), MFX-4 (330), MFX-5 (302), MFX-6 (286) |
| 15 min | CO₂ (44), H₂O (18); trace intermediates: MFX-7 (258), MFX-8 (230), MFX-9 (202) |

Note: trace = very low-intensity MS signal (≤5% relative to the base peak intensity); ND = not detected above the instrument detection limit.

Note: Sulfate, nitrate, and nitrite ions were only tentatively detected due to the limited sensitivity of ESI-MS for inorganic anions.


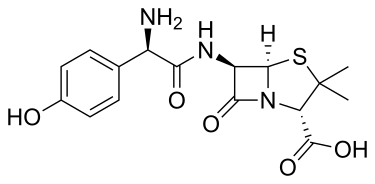


Main pathway :

AMO1 (m/z 365)

↓ (β-lactam ring cleavage → penicilloic acid derivative)

AMO-2 (m/z 170.23), AMO-3 (m/z 148.22)

↓ (oxidation, S-oxidation, deamination)

AMO-4 (m/z 175.19), AMO-5 (m/z 158.17), AMO-6 (m/z 166.13)

↓ (fragmentation of thiazolidine / side chain cleavage)

AMO-7 (m/z 112.19), AMO-8 (m/z 112.15), AMO-9 (m/z 183.13)

↓ (further oxidative fragmentation)

AMO-10 (m/z 131.18), AMO-11 (m/z 134.18), AMO-12 (m/z 167.12), AMO-13 (m/z 102.16)

↓ (final low-mass acids)

AMO-14 (m/z 60.05), AMO-15 (m/z 61.04), AMO-16 (m/z 74.08), AMO-17 (m/z 90.08), AMO-18 (m/z 82.07)

↓

CO₂ + H₂O

**Fig. S5.**Degradation pathway of amoxicillin under Co₀.₅Fe₀.₅Fe₂O₄/H₂O₂ system

**Table S5.** Detected intermediates of amoxicillin degradation at different reaction times under Co₀.₅Fe₀.₅Fe₂O₄/H₂O₂ system (MS analysis).

| **Time** | **Intermediates (m/z)** |
| --- | --- |
| 5 min | AMO1 (365), AMO-2 (170.23), AMO-3 (148.22) |
| 9 min | AMO-4 (175.19), AMO-5 (158.17), AMO-6 (166.13) |
| 12 min | AMO-7 (112.19), AMO-8 (112.15), AMO-9 (183.13), AMO-10 (131.18), AMO-11 (134.18), AMO-12 (167.12), AMO-13 (102.16), AMO-14 (60.05), AMO-15 (61.04), AMO-16 (74.08), AMO-17 (90.08), AMO-18 (82.07); |
| 15 min | CO₂ (44), H₂O (18); trace intermediates:AMO-14 (60.05), AMO-15 (61.04), AMO-16 (74.08), AMO-17 (90.08), AMO-18 (82.07), (Sulfate tentative in ESI) |

Note: trace = very low-intensity MS signal (≤5% relative to the base peak intensity); ND = not detected above the instrument detection limit.

Note: Sulfate, nitrate, and nitrite ions were only tentatively detected due to the limited sensitivity of ESI-MS for inorganic anions.


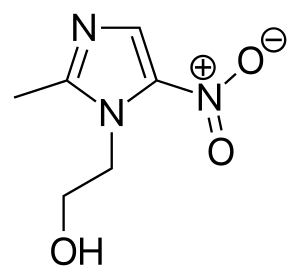


Main pathway :

MTZ1 (m/z 171.15) (parent)

↓ (N–O bond scission / nitro reduction, R-NO/R-NHOH)

MTZ-2 (m/z 128.05)

↓ (oxidative cleavage / fragmentation of imidazole ring)

MTZ-3 (m/z 85.05)

↓ (deep oxidative fragmentation)

MTZ-4 (m/z 60.05)

↓ (dehydration, minor)

MTZ-5 (m/z 42.05, trace)

↓ (further oxidation → mineralization)

CO₂ + H₂O

**Fig. S6.** Degradation pathway of metronidazole under Co₀.₅Fe₀.₅Fe₂O₄/H₂O₂ system.

**Table S6.** Detected intermediates of metronidazole degradation at different reaction times under Co₀.₅Fe₀.₅Fe₂O₄/H₂O₂ system (MS analysis).

| **Time** | **Detected species (m/z)** |
| --- | --- |
| **5 min** | MTZ1 (171.15), MTZ-2 (128.05) |
| **9 min** | MTZ-2(128.05),MTZ-3 (85.05) |
| **12 min** | MTZ-3 (85.05), MTZ-4 (m/z 60.05), MTZ-5 (m/z 42.05) |
| **15 min** | CO₂ (44), H₂O (18); trace intermediates: MTZ-4 (60.05), MTZ-5 (42.05), (Nitrate/nitrite tentative in ESI) |

Note: trace = very low-intensity MS signal (≤5% relative to the base peak intensity); ND = not detected above the instrument detection limit.

Note: Sulfate, nitrate, and nitrite ions were only tentatively detected due to the limited sensitivity of ESI-MS for inorganic anions.
